# Safety of measles, mumps, and rubella vaccine in egg allergy: in vivo and in vitro management

**DOI:** 10.1186/s12948-020-00136-3

**Published:** 2020-11-15

**Authors:** Stefania Magistà, Marcello Albanesi, Nada Chaoul, Danilo Di Bona, Elisabetta Di Leo, Eustachio Nettis, Maria Filomena Caiaffa, Luigi Macchia

**Affiliations:** 1 Department of Emergency and Organ Transplantation, School and Chair of Allergology and Clinical Immunology, University of Bari-Aldo Moro, Piazza Giulio Cesare, Policlinico, 70124 Bari, Italy; 2 Department of Medical and Surgical Sciences, School and Chair of Allergology and Clinical Immunology, University of Foggia, Via Luigi Pinto 1, 70100 Foggia, Italy

**Keywords:** Vaccine adverse reaction, Food allergy, Drug allergy, Anaphylaxis, B-cell proliferation assay, Flow cytometry

## Abstract

**Background:**

Egg allergy is the second most prevalent form of food allergy in childhood. In spite of the evidence accumulated, inoculating egg allergy children with attenuated vaccines grown on chick embryo cell cultures, such as the measles, mumps, and rubella (MMR) vaccine, is regarded (erroneously) as potentially dangerous or even anaphylactogenic, by many. An issue perceived as particularly conflicting also by Health Professionals.

**Case presentation:**

A 15-year-old boy, with a history of severe egg allergy in early infancy, who was still sensitized to egg allergens, including baked egg, had never received MMR vaccination, in fear of possible anaphylaxis, in spite of the fact that this vaccination is mandatory in the first year of life, in Italy. Because of that, he was not allowed to attend school, longer, and was referred to us in order to assess the potential risk of MMR vaccination. Upon thorough allergologic workup, sensitization to MMR vaccine components was excluded by an in vivo approach, consisting in skin prick tests, intradermal tests, and subcutaneous injection test, corroborated by vaccine-specific B-lymphocyte proliferation assay, ex vivo. T-cell proliferation in response to MMR vaccine was also excluded. Eventually, the boy was inoculated with MMR vaccine and was readmitted to school.

**Conclusions:**

The diagnostic strategy adopted appears feasible and easy-to-perform and may be adopted in controversial cases (as the one reported), characterized by previous severe allergic reactions to egg. The B-lymphocyte proliferation assay we developed may represent a useful and reliable tool not only in research but also in clinical practice.

## Background

Egg allergy may be defined as an adverse immune reaction induced by egg proteins. Egg allergy is the most common food allergy in children aged from 5 months to 15 years, following only cow milk allergy [1]. It occurs most often in early infancy, after the first egg ingestion, whereas the prevalence in adults is around 0.2% [1]. Clinical traits include urticaria, respiratory symptoms, such as change in voice pitch, cough, and wheeze, cry, pallor, gastrointestinal symptoms, such as vomit and diarrhoea, and anaphylaxis.

Twenty-four allergenic egg proteins have been identified, most of them in the egg white. Of these, ovomucoid (OVM), ovalbumin (OVA), ovotransferrin (OVT), lysozyme, and chicken serum albumin (the only major allergen present in the yolk) represent the most important egg allergens. OVM and/or OVA usually mediate type-1 hypersensitivity reactions. OVM is considered the dominant protein, from the immunological point of view, and is characterized by its stability to thermal conditions. Indeed, the immunological activity of OVM and OVA can still be detected after heat treatment: soft boiling (100 °C, for 3 min) but also hard boiling (100 °C, for 20 min). Only baking treatment (180 °C, for 20 min) is able to decrease OVM immunological activity, probably due to the higher temperature applied for a longer time or to the coupling with flour proteins, which might alter the tridimensional structure of OVM allergen [2].

Skin reactivity towards food allergens (measured by quantitative skin prick test; SPT) and food-specific IgE levels in serum (measured by RAST) are the two parameters usually used for diagnosis and management of food allergy. Taken together, they define the global levels of allergen-specific IgE, as skin reactivity is quantitatively related to the majority of IgE bound to the tissue-resident mast cells. The remaining lesser pool of IgE molecules circulates in plasma and other bodily fluids.

The measles, mumps, and rubella (MMR) vaccine is an attenuated vaccine, mandatory for all children, within the first year of age, according to the Italian legislation. The viruses used in this vaccine preparation are grown on chick embryo fibroblasts cultures.

It has been previously demonstrated that a certain amount of egg proteins is still present in MMR vaccine preparations. For this reason, it is (erroneously) believed by many that administration of MMR vaccine in egg allergy patients might lead to severe adverse reactions [3]. As a result, even some General Practitioners and Health Professionals may refuse to administer the MMR vaccine in egg allergy patients, fearing lack of safety, adding to the still conflicting issue of vaccination in egg allergy children.

## Case presentation

Hereby, we describe the case of a 15-year-old boy, diagnosed with severe egg allergy, who was referred to our Clinic because he had never received MMR vaccination, despite the fact that it was mandatory. Therefore, he could not be admitted to school, further. Indeed, his parents considered MMR vaccination highly risky because of his underlying egg allergy.

A thorough allergologic workup was carried out.

As for the clinical history, a single adverse reaction to egg was reported, which had occurred in early infancy (9 months), following the first ingestion of cooked egg. Typically, symptoms had included wheezing, dyspnea, change in voice pitch, cough, cry, and pallor. The rapid involvement of the respiratory tract indicated that the reaction was severe [4] (the child was hospitalized and treated with corticosteroids and antihistamines). After this event, eggs were completely excluded from the child diet. Moreover, when the child was 9, he suffered from anaphylactic shock, after pine nuts ingestion.

**Fig. 1:**
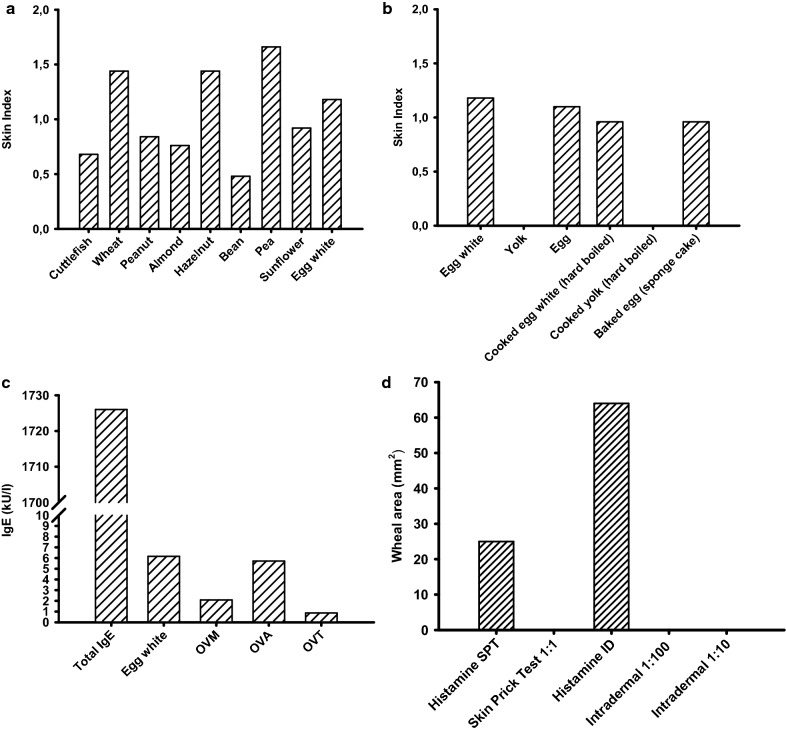
In vivo and in vitro IgE detection. **a** Quantitative SPT for a commercially available array of food allergens. Results are expressed in terms of Skin Index, i.e., the ratio between the area of the allergen wheal and the area of the exogenous histamine (10 mg/ml) reference wheal. **b** Quantitative SPT for commercially available egg extract (egg white and yolk), and prick-by-prick with cooked egg (egg white and yolk), and baked egg (sponge cake). Results expressed in terms of Skin Index, also. **c** Total serum IgE and egg allergen-specific serum IgE, as measured by ImmunoCAP. **d** Quantitative SPT with MMR vaccine (undiluted); quantitative intradermal tests with MMR vaccine, at 1:100 and 1:10 dilutions, respectively. Results are expressed as wheal areas (mm^2^). Exogenous histamine (0.002 mg/ml) was used for positive control in intradermal testing

Quantitative SPT were performed with an array of 36 commercially available food allergens (Lofarma, Milan, Italy), reflecting the spectrum of food allergy in Southern Italy (where people consume a typical Mediterranean Diet). Multiple sensitizations were detected, including peanuts, almonds, hazelnuts, wheat, and, particularly, egg white (Fig. 1a). We also performed prick-by-prick, also testing a baked cake (sponge cake; well cooked eggs) and cooked egg (hard boiled; Fig. 1b). Results were expressed in terms of ratio between the area of the allergen wheal and the area of the exogenous histamine wheal (referred to as Skin Index). Moreover, the wheals obtained with egg white, hard-boiled egg white, and baked egg were all greater than 5 mm (average diameter), regarded as associated with a high specificity in childhood [5]. These tests indicated that the boy was sensitized to multiple food allergens and that egg allergy was still present. As for the other food sensitizations detected, the young patient stated that he could eat wheat-derived foods (bread, pasta, etc.), almonds, beans, and sunflower seeds, without suffering any allergy symptoms. He had undergone modest symptoms (lip angioedema) upon occasional consumption of green peas and hazelnuts, whereas he could not remember having ever eaten peanuts, cuttlefish, octopus, and clams, of any kinds, suggesting the existence of a genuine intolerance to these foods.

Furthermore, by ImmunoCAP (Thermo Fisher Scientific, Milan), we evaluated specific IgE level in serum for egg white, OVM, OVA, and OVT. The values detected were predictive of clinically-relevant reactions, following egg consumption, confirming the skin test results (Fig. 1c). Total IgE levels were particularly high (Fig. 1c).

Having assessed egg allergy, we performed SPT with pure MMR vaccine, which proved negative. Then, we performed intradermal tests with two increasing dilutions of MMR vaccine (viz*.* 1/100, 1/10). As expected, also this procedure proved negative (Fig. 1d). Moreover, 100 µl of 1/10 dilution of MMR vaccine were injected subcutaneously (injection test). No immediate reaction was observed, either local or systemic.

**Fig. 2:**
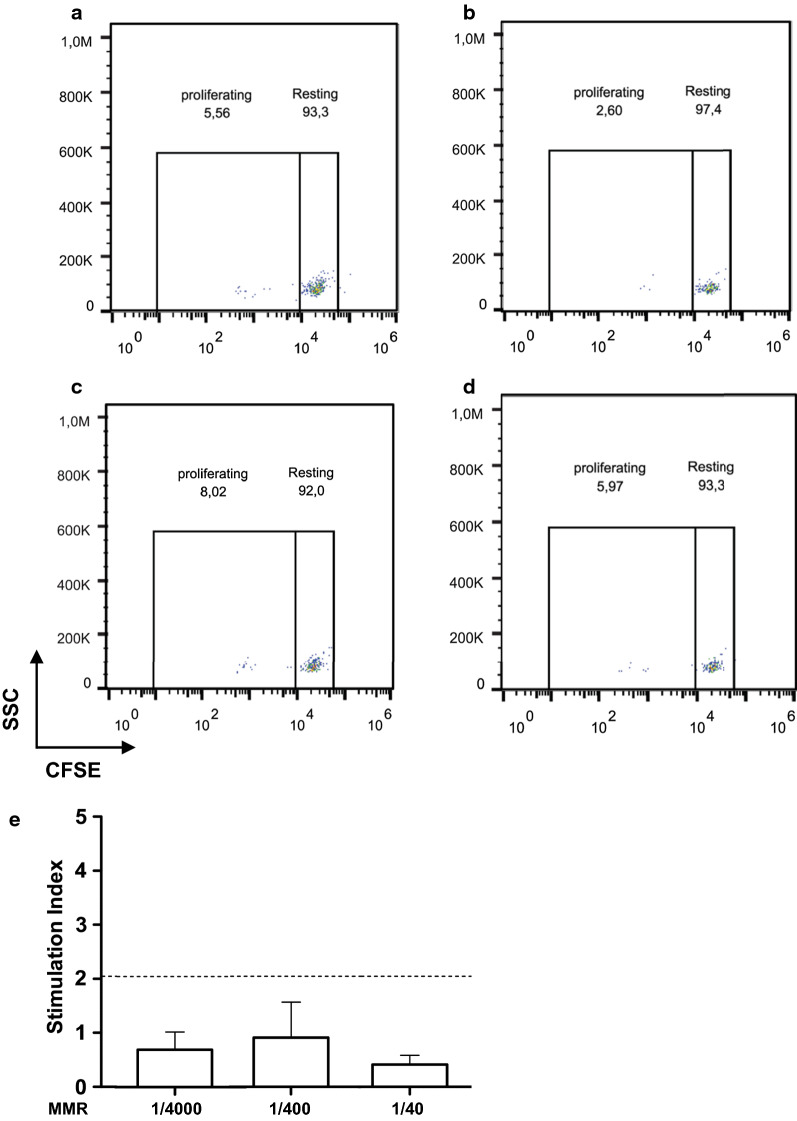
Ex vivo B-cell proliferation assay. Cell proliferation assessed by reduction of CFSE intensity: **a** in microcultures of untreated B-cells; **b** B-lymphocytes incubated with a 1:4000 MMR vaccine dilution; **c** B-lymphocytes incubated with a 1:400 MMR vaccine dilution; and, finally, **d** B-lymphocytes incubated with a 1:40 MMR vaccine dilution. Results refer to one of the 2 proliferation assays carried out. In **e** Stimulation Indexes for the 3 MMR vaccine dilutions above. Averages of the 2 assays. The Stimulation Index is the ratio between the proliferation rate of cells exposed to a potential proliferation agent and the basal proliferation rate of control cells. A Stimulation Index ≥ 2 indicates a significant specific proliferation activity. *SSC* side scatter, *CFSE* carboxyfluorescein succimydil ester

Finally, in order to confirm the absence of vaccine-specific B-cell clones, which would corroborate the results obtained in vivo, we also performed an ex-vivo B-lymphocyte proliferation assay (Fig. 2a–d) [6]. By this approach, also a possible delayed allergic response towards MMR vaccine components was investigated (proliferation of vaccine-specific T-cells). Thus, peripheral blood mononuclear cells (PBMC) were isolated as described [6] and stained with carboxyfluorescein succinimidyl ester (CFSE; 5 µM) for 5 min, washed, and cultured in Dulbecco’s modified Eagle’s medium (DMEM), supplemented with 10% of the patient’s serum. These PBMC were exposed to 3 different dilutions of the vaccine (1/4000, 1/400, and 1/40, respectively), in triplicate micro cultures (2 × 10^5^ PBMC in 200 µl), maintained at 37 °C in a 5% CO_2_, vapour-saturated atmosphere. Cultures with no vaccine addition were used as negative control. After 48 h, the PBMC were harvested, washed, and stained with fluorochrome-coupled anti-CD19 and anti-CD3 antibodies, for 20 min. After further washing, the cells were analyzed by flow cytometry (Navios 3L 10C, Beckman Coulter, Milan), for the detection of proliferating B- and T-lymphocytes. Importantly, no B-lymphocyte proliferation (CD19^+^ cells) was observed in the presence of MMR vaccine (Fig. 2a–e). CD3^+^ cell proliferation was not observed, also (data not shown).

Based on this further evidence, we administered the MMR vaccine to the boy, in two 50% doses, 250 µl each, with an interval of one hour between the two injections. The patient remained under observation for 1 h (after the second injection). As expected, no immediate nor delayed adverse reactions were observed. Therefore, he could be readmitted to school.

Nine months later, the B-lymphocyte proliferation assay was repeated with similar results (Fig. 2e). A second MMR vaccine administration was then carried out, according to the inoculation schedule specified.

## Discussion and conclusions

In this study, we describe a thorough allergy workup aimed at demonstrating in real-life that there is no association between egg allergy and possible MMR vaccine reactions, in agreement with the vast existing literature, which indicates that MMR vaccine does not contain significant amounts of detectable egg proteins (approximately 37 pg; considerably less than influenza and yellow fever vaccines) [7]. Many of the reactions described in the literature are in fact caused by gelatine, which is present at high concentration as a stabiliser [8]. Furthermore, this is the first study that proves the absence of vaccine-specific B-cell proliferation upon MMR exposure, ex vivo, in a patient with egg allergy. Although not yet fully validated, this easy-to-perform and innovative technique may be useful in the management of these cases, providing ex vivo evidence of the absence of B- and T-cell proliferation in response to MMR vaccine, prior to inoculation. Of course, other in vitro techniques, such as the basophil activation test, may also serve for this purpose.

Thus, children with egg allergy should receive their normal childhood immunizations, including MMR vaccination, as a routine procedure [9]. Only children with a documented history of anaphylaxis to egg or to MMR vaccine itself should be assessed in hospital.

## Data Availability

The clinical and laboratory records, upon which this paper is based, may be made available to third parties on request.

## Abbreviations

CFSE: Carboxyfluorescein succimydil ester
DMEM: Dulbecco’s modified Eagle’s medium
MMR: Measles, mumps, and rubella
OVM: Ovomucoid
OVA: Ovalbumin
OVT: Ovotransferrin
PBMC: Peripheral blood mononuclear cells
SSC: Side scatter
SPT: Skin prick tests
RAST: Radio allergo sorbent test
